# Inadvertent arterial & venous injury by bone marrow biopsy needle: case report on rescue embolization techniques

**DOI:** 10.1186/s42155-020-00172-9

**Published:** 2020-11-07

**Authors:** Chris Siu-Chun Tsai, Simon Chun-Ho Yu

**Affiliations:** Department of Imaging and Interventional Radiology, Prince of Wales Hospital, The Chinese University of Hong Kong, Shatin, Hong Kong SAR

**Keywords:** Therapeutic embolization, Embolotherapy, Vascular injury

## Abstract

**Background:**

Bone marrow biopsy is a common medical procedure for diagnosis and characterization of haematological diseases. It is generally regarded as a safe procedure with low rate of major complications. Inadvertent vascular injury is however an uncommon but important complication of bone marrow biopsy procedure. The knowledge of a safe and effective embolization method is crucial for interventional radiologists to reduce significant patient morbidity and mortality, shall such inadvertent vascular injury occurs.

**Case presentation:**

Bedside bone marrow biopsy was performed for an elderly gentleman to evaluate for his underlying acute leukaemia. Biopsy needle inadvertently injured the internal iliac artery and vein during the procedure. Coil embolization was carefully performed across injured arterial segment via the culprit biopsy needle until contrast cessation. Concomitant venous injury was subsequently confirmed on angiography when the needle was withdrawn for a short distance from the iliac artery. This venous injury was tackled by further withdrawing the biopsy needle to distal end of the bone marrow tract for tract embolization with coils and gelatin sponges. High caution was made to avoid coil dislodgement into the iliac vein, to prevent pulmonary embolism. Patient was clinically stable throughout the procedure. Post-procedure contrast CT shows no pelvic haematoma or contrast extravasation.

**Conclusions:**

This case illustrates rescue embolization techniques for rare life-threatening concomitant internal iliac arterial and venous injuries by a bone marrow biopsy needle. Interventional radiologists can play an important role in carrying out precise embolization to avoid significant patient morbidity and mortality in the case of life-threatening haemorrhage.

## Background

Bone marrow aspiration and biopsy is a very common diagnostic examination for haematological diseases. It carries invaluable diagnostic and prognostic implications. In our local clinical setting, bone marrow biopsy is often a bedside procedure performed by junior doctors. It has remained a relatively safe procedure with few complications such as wound site oozing, local infection, needle fractures and neurovascular injury (Bain 2005, 2006; Fisher 1971). Fatalities from vascular injury are rare, though have been reported in the literature (Chamisa 2007; Gupta et al. 1992).

## Case presentation

A 70 years-old male patient presented to the Accident & Emergency Department for increasing shortness of breath and extremity edema. Subsequent blood tests revealed marked leucocytosis (WBC 317.6 × 10^9/L) and high percentage of blast cells on peripheral blood smear. The diagnosis of acute leukaemia was made.

Bone marrow biopsy was arranged at bedside after admission. Biopsy was planned at the posterior superior iliac spine (PSIS) of the left iliac bone with a 11-Gauge Jamshidi bone biopsy needle (Carefusion, USA). Needle was advanced into the left iliac bone gradually with a sudden give-way. Arterial spurting was observed from the needle hub upon removal of the needle stylet. At this juncture, arterial injury was suspected and stylet was immediately reinserted to stop the arterial bleeding. Patient was clinically stable.

Urgent contrast-enhanced CT was performed for anatomical delineation, which showed that the biopsy needle punctured the left internal iliac artery distal to the take-off of the left obturator artery with no adjacent haematoma.

**Fig. 1:**
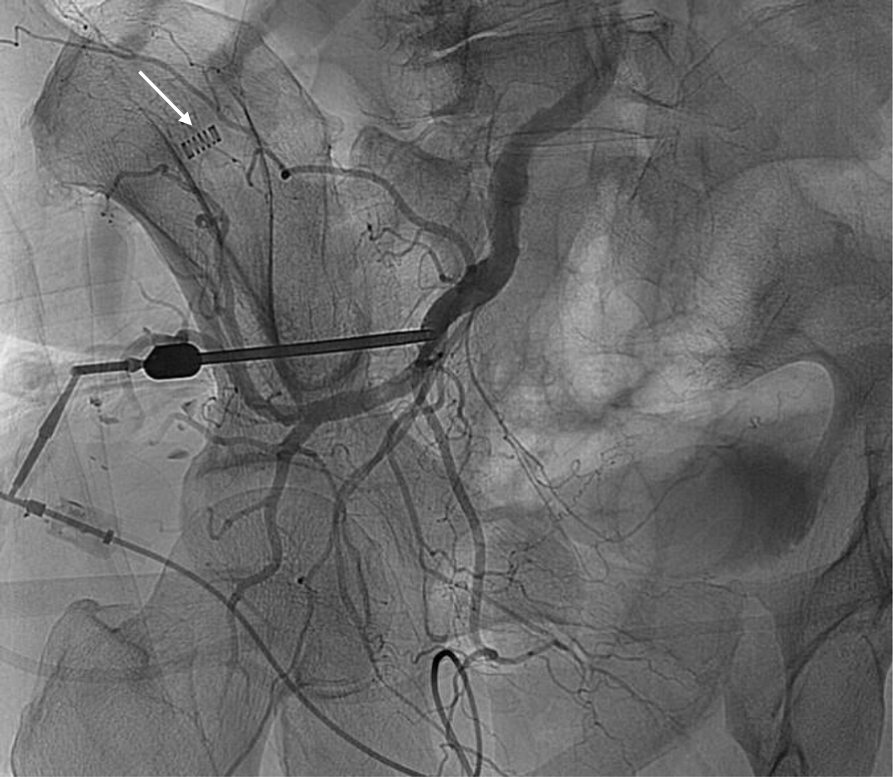
Direct needle angiogram via the bone marrow biopsy needle showed opacification of the left internal iliac artery. A springe-like radio-opacity (white arrow) which belonged to an extracorporeal tubing was noted

Patient was immediately transferred to angiography suite for rescue embolization. Since the needle was still left in the left iliac bone and any excessive movement could dislodge the needle and resulting in life-threatening haemorrhage, patient had to remain in prone position for the procedure. A Y-shape adaptor with hemostatic valve was applied to the hub of the Jamshidi needle after removal of the needle stylet. Angiogram performed via the needle shows contrast opacification of the left iliac artery and its distal branches (Fig. 1). The bevel edge of the needle was pointing superiorly. Decision was made at this juncture for coil embolization of the injured arterial segment. 5Fr RC-1 catheter (Cook Medical, USA) was inserted via the biopsy needle and aligned with the needle tip. Pushable Nester embolization coils 8mmX4 and 14mmX5 (Cook Medical, USA) were deployed which occluded the segment of internal iliac artery above the puncture site (as the coils follow the superiorly-pointing bevel of the Jamshidi needle). The segment of internal iliac artery inferior to the puncture site was embolized as well to prevent back-bleeding using detachable Interlock Coils (Four 6mmX20cm coils and one 10mmX30cm coil) (Boston Scientific, USA), which were placed through a 2.5Fr Renegade microcatheter (Boston Scientific, USA). Control angiogram via the Jamshidi Needle showed no contrast flow at the embolized segment.

**Fig. 2:**
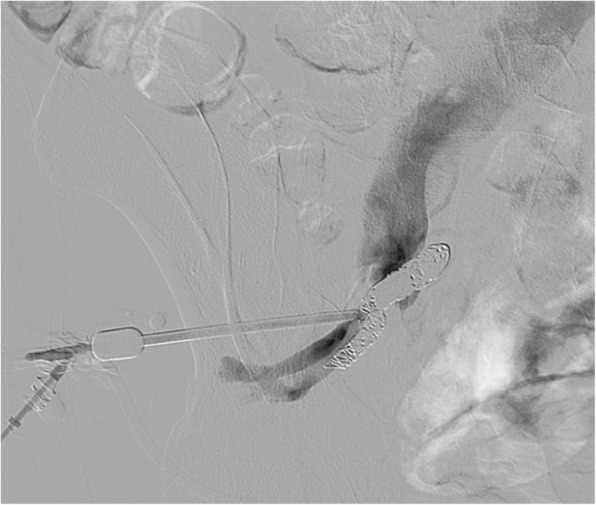
Direct needle angiogram showed contrast outlining the left iliac vein and lower inferior vena cava, thus confirming concomitant venous injury

With apparent success of the arterial embolization, the biopsy needle was slowly withdrawn. Active oozing from needle hub was noted when the needle was pulled out for approximately 1 cm. The Y-shape adapter was immediately secured back onto the needle hub. Angiogram was again performed which outlined the left iliac vein with opacification of the lower IVC (Fig. 2). This confirmed concomitant left iliac venous injury.

**Fig. 3:**
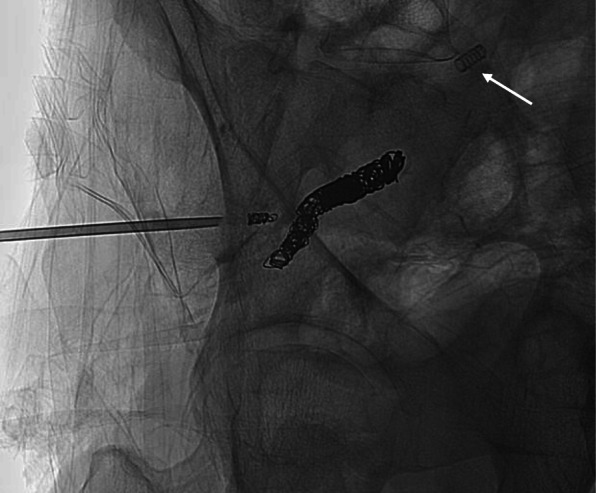
Further embolization of the bone marrow tract with coils and gelatin sponges. The same spring-like radio-opacity was again noted (white arrow) in reference to the pre-embolization angiogram, not to be confused with coil migration

The Jamshidi needle was slowly pulled out just away from the venous lumen. Similar blood oozing was noted from the needle hub. This confirmed that there was no collapsible soft tissue between the injured vein and iliac bone. Thus, the injured vein was very likely directly abutting onto the iliac bone. At 8 mm from the venous wound, another angiogram was performed which outlined a bony canal connecting directly to the injured vein without extravasation. The bony canal was embolized with a 3 mm × 12 cm Interlock coil using a Direxion 021 microcatheter (Boston Scientific, USA). The needle track outside the bony canal was embolized with 3 cc gelatin sponges after further withdrawal of the Jamshidi needle for 5 mm (Fig. 3). Further gradual withdrawal of the needle showed no more oozing and needle was completely removed.

Patient was stable throughout the procedure. Post-embolization CT shows no contrast extravasation nor pelvic hematoma.

## Discussion & Conclusions

This present case illustrates a safe and practical embolization technique to avoid life-threatening haemorrhage in inadvertent vascular injury caused by bone biopsy needle.

Pulsatile arterial spurting from the Jamshidi needle hub indicates high likelihood of arterial injury. Immediate reintroduction of the needle stylet is crucial to tamponade the haemorrhage. Often the patient is in prone position in this scenario (PSIS targeted). In such position, this is not preferred to insert a sheath in the common femoral artery. Popliteal artery catheterization is an alternative, by which however, concomitant venous injury could not be tackled. Thus, the culprit needle is the preferred venue for embolization. This case showed it is safe to perform embolization through the needle without dislodgment of the needle tip from the artery during embolization, because the needle is securely fixed by the pelvic bone.

Coil embolization was adopted in the current case, with deployment across the injured arterial segment. F. Chu et al. (2018) described another method of embolization with absorbable gelatin sponges followed by n-butyl cyanoacrylate (NBCA) in a similar scenario. The use of embolization coils is preferred because it allows precise occlusion of the injured artery without a risk of pelvic tissue ischaemia from unpredictable distal migration of gelatin sponges or NBCA.

To add to the complexity, venous bleeding was encountered upon slow needle withdrawal after treatment of the arterial injury. Embolization of the iliac vein was considered contraindicated because of the risk of deep vein thrombosis and pulmonary embolism. Removal of the needle without embolization might lead to blood loss and hematoma formation. The authors decided to embolize the needle track close to the venous wound. The use of gelatin sponges in addition to embolization coils was to facilitate immediate hemostasis which was confirmed on delayed post-embolization CT scan.

In conclusion, given the prevalence of bedside bone marrow biopsy procedure, it is important for interventional radiologists to be aware of potential rescue method, as demonstrated by this case, to avoid significant patient morbidity and mortality.

## Data Availability

Not applicable.

## Abbreviations

PSIS: Posterior Superior Iliac Spine
